# Bibliographical

**Published:** 1894-03

**Authors:** 


### Bibliographical.

Popular Essays on the Care of the Teeth and Mouth, by Victor C. Bell, A B., D.D.S., Published by the Author. 1894.

This is a little work "prepared in the hope that it may in some way be used to spread a knowledge of the importance of the dental organs among the people, especially the young, through the medium of the schools, and through its teachings, if well followed, much of pain and suffering would be prevented and the general term of human life be perceptibly lengthened. It has not been written especially for dentists but chiefly for the public."

The work consists of twelve chapters and about one hundred pages. It discusses briefly and quite clearly, Cleanliness of the Teeth, Filling, Extraction, Artificial Teeth, Children's Teeth, Crown and Bridgework, Advice to Mothers, etc., in a way that cannot be otherwise than profitable to the general reader. To give information upon these sunjects to the public has long been regarded by the profession as a very important matter. Were the people properly informed of the value of the teeth to the animal economy, and what can be done for their preservation, and be made sufficiently to appreciate them in all respects, there would be vastly less of disease of the mouth and teeth and loss of the latter than is now realized.

This work if it could be distributed to the public would certainly do much towards furthering a knowledge of and appreciation for the teeth. If it were possible to put it in a cheaper form, so that it could be purchased by dentists and distributed to their patients, its influence would be far more widespread. There have been quite a number of productions of this kind prepared and distributed, and doubtless each has had its influence. But while such works are desirable and may accomplish much good, yet they cannot take the place of the instruction which the well equipped dentist can give personally to his patients. The ability to do this ought to be cultivated by every student preparing himself for dental practice.

Dr. Bell will doubtless have the thanks of the profession for this book, and we would heartily commend it to every dentist for use in his reception room.

